# Physiological adjustments of temperate tree species and herbs in response to low root temperatures

**DOI:** 10.1093/treephys/tpaf018

**Published:** 2025-02-04

**Authors:** Yating Li, Guenter Hoch

**Affiliations:** Department of Environmental Sciences-Botany, University of Basel, Schönbeinstrasse 6, Basel 4056, Switzerland; Department of Environmental Sciences-Botany, University of Basel, Schönbeinstrasse 6, Basel 4056, Switzerland

**Keywords:** cold acclimatization, growth limitation, hydraulic constraints, water relation

## Abstract

Hydraulic constraints induced by low root temperature might be a major cause for the low temperature limit of plants. However, to date most of our knowledge on the physiological effects of low root temperatures is derived from short-term lab experiments, with very limited information on potential adjustments to continuous low temperature stress. In this study, we quantified the cold sensitivity of root water uptake and transport to leaves in seedlings of different functional plant types (conifers, broadleaved trees and annual herbs) by ^2^H-H_2_O labeling after exposure to three constant root temperatures (15 °C, 7 °C and 2 °C) but the same higher aboveground temperatures (between 20 and 25 °C). We investigated changes in the cold sensitivity of roots after 0, 10 and 20 days prolonged exposure to the respective root temperatures. Plant water uptake and transport was decreased by lowered root temperature in all species, with a stronger effect at 2 °C compared with 7 °C. The water uptake and transport capacity of tree species gradually declined over the 20-day treatment, while the two investigated herbs exhibited immediately strong decreases that were kept at the same low level throughout the entire experiment time. The speed of the water uptake reduction across the 20 days observation period differed among the tree species and was faster in species that reach their natural upper distribution limits at lower elevations compared with species that occur at subalpine regions. The restricted root water uptake and transport was accompanied by reductions in leaf water potential, stomatal conductance and growth. Overall, our study showed increasingly reduced capacity for water uptake and transport across functional plant groups at continuous cold root conditions. This result might indicate accumulative negative effects on cell membrane permeability for water in roots, or a controlled reduction of root water conductivity of temperate trees in preparation for winter dormancy.

## Introduction

Low temperature is an essential abiotic stress affecting plant phenology, growth, reproduction and thus determining species distribution range limits (Woodward 1990, Morin et al. 2007, Körner 2021). In general, plants are able to anticipate ambient temperature fluctuations and mitigate or avoid negative effects of cold temperatures through physiological and metabolic adjustments (Beck et al. 2007, Nievola et al. 2017). Low root temperatures have been identified as being especially relevant to plant productivity, since they can severely restrict root water uptake already well above 0 °C, with negative effects on plant hydration and growth (Wan et al. 1999, Nagasuga et al. 2011, Schenker et al. 2014). However, our current knowledge on the negative effects of low root temperatures on plant-water relations has mainly been derived from short-term experiments over minutes to hours (Arndt 1937, Ameglio et al. 1990, Wan et al. 2001, Bloom et al. 2004, Lee et al. 2005), while little is known so far about the ability of plants to adjust physiologically to low root temperatures over longer observation periods.

Already very early plant-physiological experiments (with the first published observations actually dating back to the early 18th century, Hales 1727) revealed the adverse effects of low, but non-freezing root temperature on the hydraulic status of plants, as indicated by plant wilting below species-specific root temperature thresholds (Clements and Martin 1934, Arndt 1937, Sachs 1875, Vesque 1878, Kuiper 1964). These studies suggested low root temperature-induced drought-like water deficits caused by low root temperatures that lead to reduced cell turgor pressure and consequent plant wilting (Bagnall et al. 1983, Pollock et al. 1990, Beck et al. 2007). Further studies confirmed that the low temperature sensitivity of root water uptake varies among plants species, and that the species-specific sensitivity in plant water uptake against decreasing temperatures can be assessed as the critical break-point temperature in Arrhenius plots, where a dramatic reduction of water uptake can be observed above 10 °C for chilling-sensitive herbs (Merrill 1975, Bloom et al. 2004, Lee and Chung 2005, Murai-Hatano et al. 2008), ⁓10 °C for chilling-tolerant herbs and temperate tree species (Kramer 1942, Kuiper 1964, Kaufmann 1975, Wan et al. 2001), and below 5  °C for boreal tree species (Kramer 1942, Grossnickle 1988, Dang and Cheng 2004). However, even cold-adapted boreal and montane tree species showed severe limitations of root water uptake at root temperarures between 5 and 0 °C (Running and Reid 1980, Li and Hoch 2024, Wang and Hoch 2022).

Many plant-physiological changes induced by chilling soil temperatures are generally reversible processes which has been documented not only in different herbs (Ameglio et al. 1990, Fennell and Markhart 1998, Melkonian et al. 2004) but also in boreal and temperate trees (Delucia 1986, Day et al. 1991, Lippu and Puttonen 1991, Mellander et al. 2004, Lintunen et al. 2020). Several studies confirmed restricted hydraulic relations at low soil temperatures also in mature trees under field conditions (Mellander et al. 2004, Wieser et al. 2015, Yan et al. 2019, Lintunen et al. 2020). Low temperature below a species-specific threshold results in a dramatic decline in plant root hydraulics due to the restricted symplastic water transport in roots with additional negative effects from increasing water viscosity (Kramer 1942, Kaufmann 1975, Ameglio et al. 1990, Cochard et al. 2000, Wan et al. 2001), subsequently resulting in the reduction of leaf water potential, stomatal conductance and photosynthesis (Lippu and Puttonen 1991, Lintunen et al. 2018, 2020), also in situations of low root but relatively warmer shoot temperatures (Wang and Hoch 2022). Such negative effects of low root temperatures on plant hydration might be an important factor limiting the expansion of growing cells, resulting in a restriction of new tissue formation and plant growth (Cosgrove 1993, Nagelmüller et al. 2017, Peters et al. 2021), consequently contributing to the cold limits of plant distribution (Li and Hoch 2024).

Numerous studies have investigated low temperature effects on root hydraulic conductance in experimental observations at controlled root temperatures with herbs (Ameglio et al. 1990, Fennell and Markhart 1998, Bloom et al. 2004, Lee et al. 2004) and tree seedlings (Running and Reid 1980, Wan et al. 2001, Wang and Hoch 2022). The majority of those previous studies investigated plants at a single time point (hours to days) after the start of the temperature treatment. Dynamic adjustemnts of plant water uptake and conductivity were mainly studies only over very short time periods of hours to a view days. One exemption is a study by Wan et al. (1999), who followed changes of water relations in response to continuous root cooling in *Populus tremuloides* seedlings over 28 days. This study indicated no or very limited acclimation of water uptake over time (Wan et al. 1999). However, we are still largely lacking systematic investigations of potential changes in root water uptake capacity and subsequently physiological adjustment to continuous chilling stress over longer time periods, especially across different functional plant groups. Such studies would be also helpful to improve models for plant growth and leaf gas exchange that currently do not consider cold soil effects on plant hydration (Lintunen et al. 2020, Zhu et al. 2021, Liu et al. 2024).

In this study, we applied deuterium (^2^H)-H_2_O pulse-labeling to directly identify the speed of water uptake and transport in different functional plant species (three conifer, seven broadleaved tree and two herbaceous species) in response to three constant root temperatures (15 °C, 7 °C and 2 °C) but the same warmer aboveground temperatures (between 20 and 25 °C) after a 0, 10 and 20 days acclimatization period, respectively. We synchronously also measured leaf water potentials of all species and stomatal conductance of broadleaved trees and herbs, and assessed relative growth rates of seven out of the 12 studied tree species after 20 days exposure to the different root temperatures. With this study, we aimed to assess the following hypotheses (i) Along the 20-day treatment period, plants show adjustments to low root temperatures that result in increasing water uptake and transport with exposure time to low root temperatures. (ii) There are significant differences among functional plant groups with respect to the cold sensitivity of root water uptake and transport and the acclimation potential to cold root temperatures. (iii) The ability of a tree species to acclimatize to low root temperatures correlates positively with its natural upper distribution limit.

## Materials and methods

### Plant material

In this study, we investigated 10 European temperate tree species including seven angiosperm and three conifer species (Table 1). In addition, we compared the tree species with two annual, relatively cold-sensitive herbaceous species (*Zea mays* L. and *Helianthus annuus* L.). The tree species were selected to cover a broad spectrum of temperature preferences according to their upper elevational distribution limits (Table 1). The species-specific upper natural distribution limit for each investigated species is here given as the relative thermal distance (in Kelvin) to the alpine treeline, following the concept by Randin et al. (2013) and assuming a mean altitudinal temperature lapse rate of 0.55 K per 100 elevational meters. Thus, the lower the thermal distance to treeline of a species, the higher its elevational range limit. All values for the tree species’ thermal distances to treeline derived directly from Li and Hoch (2024), who also provide a detailed description of the underlying tree distribution data base and the used models. Seeds of all tree species used for the experiment were acquired from the nursery of the Swiss Federal Research Institute for Forest, Snow and Landscape (WSL, Birmensdorf, Switzerland). Although we always selected the highest available provenance for each tree species, we had no access to seed material from provenances directly at the high elevation limit of the respective species. The seeds of the two annual herbs were purchased from a local seed retailer (SelectSamen, Switzerland). Table 1 summarizes alle investigated species and the provenances of all tree species.

**Table 1:** List of the investigated species. For tree species, the natural thermal distance to treeline, the seed collection site (provenance) and the elevation of the provenance are indicated. For all species, the date for seed germination and the starting date for the cold root treatment, as well as information on individual labeling dates and growth analyses are given.

Species	Thermal distance to treeline (K)	Functional plant type	Provenance (collection site)	Elevation of collection site (m a.s.l.)	Date for seeds germination (DD/MM/YYYY)	Start date for cold acclimation in water bath (DD/MM/YYYY)	Treatment period (day) included in isotopic labeling (Yes/No)			Species used for growth analyses (Yes/No)
							0	10	20	
*Alnus glutinosa*	7.3	Broadleaved tree	Wettswil Fischbach	470–560	02.07.2020	17.08.2020	Yes	Yes	Yes	Yes
*Pinus sylvestris*	5.5	Conifer	Schward Spl. Lilliental	800–900	04.02.2022	31.03.2022	Yes	Yes	Yes	Yes
*Malus sylvestris*	3.5	Broadleaved tree	Mittelland HG5	380–550	15.03.2021	02.05.2021	Yes	Yes	Yes	Yes
*Fagus sylvatica*	3	Broadleaved tree	Allschwil	381	04.02.2021	09.03.2021	Yes	Yes	Yes	Yes
*Alnus viridis*	−0.25	Broadleaved tree	Wassen	1800–2000	28.06.2020	07.08.2020	Yes	Yes	Yes	Yes
*Picea abies*	−0.25	Conifer	Birmensdorf	380	31.05.2020	27.07.2020	Yes	Yes	Yes	Yes
*Pinus nigra*	7.75	Conifer	Leuk	980–1250	25.05.2020	06.07.2020	Yes	Yes	Yes	Yes
*Quercus robur*	7.35	Broadleaved tree	Oberwil	365	10.02.2022	22.03.2021	Yes	Yes	No	No
*Tilia cordata*	5.25	Broadleaved tree	Allmeind	460–640	31.12.2020	02.02.2021	Yes	No	Yes	Yes
*Ulmus minor*	8	Broadleaved tree	Dotzigen Euchubach	430	24.11.2020	31.12.2020	Yes	Yes	Yes	Yes
*Helianthus annuus*		Annual herb	/	/	24.05.2021	10.06.2021	Yes	Yes	Yes	No
*Zea mays*		Annual herb	/	/	15.03.2021	03.03.2021	Yes	Yes	Yes	No

### Experimental set-up

The experiments were conducted in the greenhouse of the University of Basel, Switzerland between February 2020 and April 2022 with different periods for the individual experiments across the investigated species (the specific dates of seeds germination and cold acclimation for individual species are shown in Table 1). Because of the large number of seedlings, the experiments were performed in separate patches as listed in Table 1. The climate in the greenhouse was kept at 24.8 ± 2.3 °C daytime and 21.7 ± 2.6 °C nighttime air temperatures, and 50–68% (average 57.3 ± 8%) relative humidity throughout the experiment. The greenhouse was equipped with lamps to increase the natural sunlight on overcast days and keep a constant day-length of 14 h for all experiment patches. In addition, the transparent roof was automatically covered by sunshade nets during daytime at hot and very bright sunny days to avoid too strong radiation and warming. All seeds for the experiments were germinated in the greenhouse in germination trays (50 × 30 × 8 cm) filled with a 1:1 mixture of two commercial substrates: a container soil with 260 mg L^−1^ nitrogen, 180 mg L^−1^ phosphate and 480 mg L^−1^ potassium (Ökohum, Herbertingen, Germany), and a cultivation soil with 40 mg L^−1^ nitrogen, 80 mg L^−1^ phosphate and 400 mg L^−1^ potassium (Ökohum, Herbertingen, Germany). To consecutively provide sufficient space for each seedling, five to six seedlings were respectively transplanted into (8 × 8 × 8 cm) pots filled with the germination substrate a few days after germination. All seedlings were watered daily with tap water until the start of the experiment.

For our experiment, we used 1.5- to 2-month-old seedlings for all trees and 20-day-old seedlings for the two herb species. Within each species, we selected only seedlings with similar size and specific criteria for each functional plant type: ca 5 cm height for conifers, four developed leaves for broadleaved trees and *H. annuus*, and three leaves for *Z. mays*. Before the transfer to the water baths, all seedlings were carefully uprooted and gently rinsed from adhering substrate particles on the roots by tap water. Afterwards, each naked-rooted seedling was rapidly transferred into a 50 ml tube (round opening with 3 cm diameter, Falcon, BD Biosciences, Bedford, MA, USA) filled with tap water (pH = 7.03) and fixed with a round, 1.5 cm thick sponge in the center of tubes with roots completely submerged under water. For each species, ⁓200 seedlings were randomly transferred to one of three water baths with different constant water temperatures (2 °C, 7 °C and 15  °C). The seedlings were exposed to the respective root temperature for either 0 (0.5 h), 10 or 20 days before the isotopic pulse labeling was applied to quantify water uptake speed. All hydroponic seedlings were prepared in advance and left outside of the water bath at least 12 h before the start of root temperature treatments.﻿ We daily refilled fresh tap water of hydroponic tubes to refill water loss from transpiration and to avoid root hypoxia (lack of oxygen) in the hydroponic water. No additional nutrients were applied to the hydroponic since we anticipated to keep the same growth condition throughout the 20-day acclimatization, and we did not expect significant nutrient limitation in this short time period considering that the roots were only lightly rinsed and tap water was used for the hydroponic setup.

To control and manipulate the root zone temperature of the hydroponic seedlings we used the same water-bath system as described in Li and Hoch (2024). Each of three double-walled stainless-steel water baths (inside dimensions: 80 × 60 × 20 cm) was connected with a separate thermostat (Heto-Holten CBN 28-30, Allerød, Denmark) that circulated a water based anti-freeze solution through a tubing-system at the bottom of the water baths. Each water bath was filled with deionized water, and the water tables of the water baths were adjusted to sit at the water level of the hydroponic tubes. The water temperature inside of three water baths, were individually set to three constant target temperatures: 15 °C, 7 °C and 2 °C, respectively. To place the hydroponic tubes with the seedlings in the water baths, we installed plastic racks to the bottom of the water baths. The water inside the water baths was isolated from the air by a 15 mm thick rubber sponge above the water surface, which restricted the temperature exchange between the water and the air above the water surface, and reduced the average daily temperature amplitude of the water to < 1 K for each target temperature. In addition, a large fan was installed at the side of the water baths to avoid still air pockets above the sponge surface and provide the same air temperatures for all investigated seedlings. The water baths were activated at least 1 week before the individual experiments and the achieved water temperatures were controlled and adjusted daily for setting accurate water temperatures. 

### Isotope labeling

Pulse labeling with deuterium (^2^H) enriched source water was applied to study the rate of water uptake and transport from roots to leaves in all investigated seedlings treated with different root temperatures. A ^2^H-H_2_O stock solution was prepared by mixing 0.163 mL of 99.6% deuterated water with 1800 mL tap water resulting in a 500‰ δ^2^H water stock that was used for the pulse labeling. The labeling was initiated by replacing the entire water within a hydroponic tube with the 500‰ δ^2^H stock solution between 8:40 and 9:40 a.m. To investigate potential acclimation to low root zone temperatures, ^2^H labeling was performed on a subset of the seedlings at three time points: 30 min after transfer of the seedlings to the respective water bath temperatures (0 days treatment), and in the morning of the 10th and 20th day after transfer (10-day and 20-day treatment, respectively). For each species and time point, at least 10 seedlings were labeled simultaneously within each water bath. At least five seedlings per species (*n* = 5) of each water baths were randomly harvested at the following labeling time: 0.5 h before pulse-labeling (i.e., unlabelled controls), 2 h and 6 h after labeling. At harvest, all leaves or needles of each seedling were separately collected into airtight exetainers (Labco, Lampeter Credington, UK) that were sealed with a screw-cap. All exetainer caps were additionally sealed with parafilm to avoid any loss of water vapor from the exetainers and immediately stored in a freezer at −20 °C until leaf water extraction.

### Physiological measurements

On the days of ^2^H-H_2_O labeling (i.e., 0, 10 and 20 days after transfer to the water baths), leaf water potential (in MPa) was determined for all species at midday (12:30 p.m. to 2:30 p.m.) at least in five seedlings of each species per water bath with a Scholander pressure chamber (PMS 1000, PMS Instrument company, Albany, OR, USA). Because measurements were performed on unshaded seedlings, we assume that the measured water potentials were at or close to the dial minimum values (i.e., the diurnal maximum strain on the seedlings water conducting system). At the same day, the stomatal conductance (in mmol H_2_O m^−2^ s^−1^) of broadleaved species and herbs were measured with a leaf porometer (SC-1, Decagon Devices, Pullman, WA, USA) at least in five individuals of each species per water bath between 10:00 a.m. and 12:00 p.m. We did not measure the stomatal conductance of conifers because the small size of investigated seedlings.

### Biomass and relative growth rate measurements

After harvest, the roots and stems of individual seedlings that were used for pulse labeling were separately collected in small paper bags and dried at 80 °C in a drying oven for at least 48 h before weighing for dry biomass. Additionally, we weighed the total dry mass of leaves or needles after the water extraction for ^2^H analyses of leaf water. The biomass was analyzed only for a subset of the investigated tree species (four broadleaved and three conifer tree species, Table 1).

The relative growth rate (g g^−1^ day^−1^) of each organ (roots, stem, leaves) and whole plant was calculated separately as ${\log}_e\frac{\mathrm{dry}\ \mathrm{biomass}\ \mathrm{at}\ 20\ \mathrm{day}\mathrm{s}\ }{\mathrm{dry}\ \mathrm{biomass}\ \mathrm{at}\ 0\ \mathrm{day}\ }$ and then divided by the number of days (20) at root temperature treatments, referred to the method of Fletcher et al. (2022). Here, the dry biomass (0/20 days) is averaged values across seven measured tree species. Finally, to investigate the relative effect of root temperature on the root:shoot ratio, we calculated the ratio of total aboveground to total root dry biomass for day 0 and day 20 of the experiment.

### Isotopic analyses and calculations

Leaf water was extracted by the cryogenic distillation method described in Li and Hoch (2024). The leaf samples within the exetainers were heated for 3 h to 90 °C in a water bath under an applied vacuum of 0.03 hPa to completely evaporate all leaf water which was collected in U-tube glasses submerged in liquid nitrogen. After thawing, the collected water samples were transferred to 1.5 mL air-tight GC vials (Macherey-Nagel GmbH, Düren, Germany) with a syringe for isotopic analysis.

The analyses of the hydrogen isotopic composition of extracted leaf water were carried out at the Stable Isotope Ecology Lab, at the Department of Environmental Sciences, University of Basel as described in detail in Li and Hoch (2024). In short, the water molecules were converted to H_2_ and CO gasses at 1400 °C by a high-temperature conversion/elemental analyzer (TC/EA) coupled (via a Conflo IV interface) with a DeltaPlus V continuous flow isotope ratio mass spectrometer (IRMS, Thermo Fisher Scientific, Bremen, Germany). To ensure the consistency of observations, we regularly corrected the measurement errors caused by instrument drift according to the specifications and standardized the data. In this study, the δ^2^H notation describes the ^2^H content of leaf water in ‰ according to the VSMOW-SLAP standard (Vienna Standard Mean Ocean Water, and Standard Light Antarctic Precipitation, respectively), which was expressed as:


(1) \begin{equation*} {\mathrm{\delta}}^2\mathrm{H}=\left(\frac{{\mathrm{R}}_{\mathrm{sample}}}{\kern0.50em {\mathrm{R}}_{\mathrm{standard}}}-1\right) \end{equation*}

Where R_sample_ is the ^2^H/^1^H isotope ratio of plant samples, R_standard_ is the standard ^2^H/^1^H isotope ratio. Long term analytical precision for δ^2^H analyses on water samples in the lab is tracked by repeated analyses of a quality control sample, and is 0.6 ‰.

The increase of ^2^H in plant material was expressed as $\Delta{}{}^2\mathrm{H}$ notation after pulse-labeling. Basically, the amount of isotope tracer was larger than those of natural abundance after labeling, which was expressed as big delta (Δ). Therefore, the $\Delta{}{}^2\mathrm{H}$ in leaf water showed the differences between a plant sampled enriched deuterium isotope and a specific-species baseline, which can be represented as:


(2) \begin{align*} \Delta{}{}^2\mathrm{H}=\frac{\mathrm{\delta}^2{\mathrm{H}}_{\mathrm{labeled}}-{\mathrm{\delta}}^2{\mathrm{H}}_{\mathrm{unlabelled}}}{1+{\mathrm{\delta}}^2{\mathrm{H}}_{\mathrm{unlabelled}}} \end{align*}

where, ${\mathrm{\delta}}^2{\mathrm{H}}_{\mathrm{labeled}}$is the δ values of leaf water of enrichment ^2^H plant samples (labeled samples), and ${\mathrm{\delta}}^2{\mathrm{H}}_{\mathrm{unlabelled}}$ is the δ values of leaf water without ^2^H labels in seedlings sampled before the start of the ^2^H-labeling.

### Data analysis and statistics

To quantify the relative difference of water uptake and transport at 7 °C and 2 °C root temperature compared with 15 °C root temperature, we calculated the change in $\Delta$^2^H at 2 °C or 7 °C relative to 15 °C (in %) as follows:


(3) \begin{align*}\kern-.5pc\mathrm{Relative}\ \mathrm{change}\ \mathrm{in}\ \Delta^{2}\mathrm{H}= \frac{\Delta ^2{\textrm{H at}}\ {2}^{{}^{\circ}}\mathrm{C}\ {\textrm{or}}\ {7}^{{}^{\circ}}{\textrm{C}}}{\Delta ^2{\textrm{H at}}\ {15}^{{}^{\circ}}\mathrm{C}} \times 100\% \end{align*}

where $\Delta$^2^H at 2 °C, 7 °C and 15 °C are the $\Delta$^2^H values of each investigated seedlings exposed to the respective root temperature 6 h after the start of the isotopic labeling. The relative change in water potential and stomatal conductance (in % relative to 15 °C root temperature) were also calculated accordingly.

The data of $\Delta$^2^H, water potential and stomatal conductance in all tree species after 10 days acclimation presented in this study are a subset of 16 tree species that had been previously published in Li and Hoch (2024). The effect of lowered root temperatures on root water uptake indicated by $\Delta$^2^H labels in leaf water, water potential, stomatal conductance, biomass, relative growth rate and the ratio of shoot to root was tested for significance separately for each species by least significant difference tests (LSD.TEST, *P <* 0.05). Additionally, least significant difference tests (LSD.TEST, *P <* 0.05) were used to test for significant differences among the functional plant types (broadleaved trees, conifers, herbs). The significance of average growth rate of different plant organs by exposure to the same root temperatures was tested using Tukey HSD Test (rstatix) where the significance levels were indicated by ^*^(*P <* 0.05), ^**^(*P <* 0.01) and ^***^(*P <* 0.001). Linear correlation was used to analyze the dependency of the relative change of water uptake rates and water potential at 7 °C and 2  °C root temperature with the species’ thermal distances to treeline after 0, 10 and 20 days acclimatization. The effects of species, root temperature, acclimatization duration (days) and their interaction on relative change in $\Delta$^2^H, water potential and stomatal conductance were tested for significance using full-factorial ANOVA tests. All statistical analyses were carried out using R v 4.2.0 (R Core Team 2022).

## Results

### Changes of water uptake, leaf water potential and stomatal conductance with exposure time to low root temperatures

**Figure 1:**
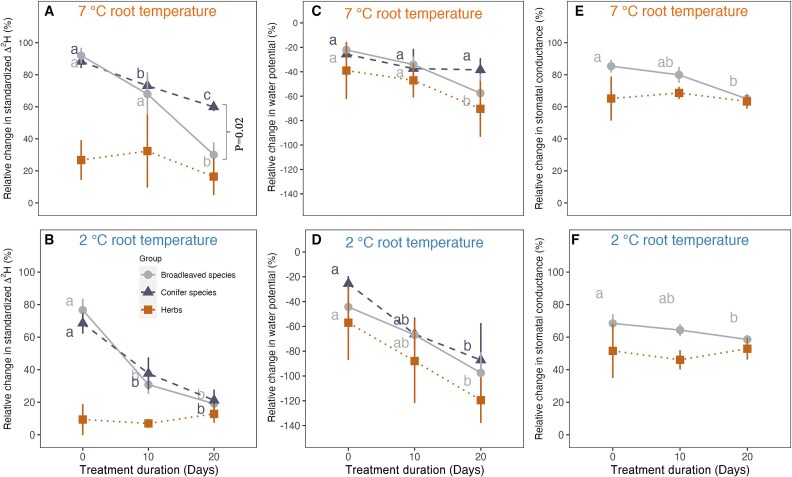
Average relative change in Δ^2^H in leaf water, leaf water potential and stomatal conductance across three functional types (conifers, broadleaved tree species and herbs) of seedlings exposed to 7 °C (A, C, E) and 2 °C (B, D, F) relative to 15 °C root temperature along treatment duration (days). The species number of different functional types types can be found along treatment duration in Figures S1 and S2 available as Supplementary data at *Tree Physiology* Online. Different lowercase letters represent significant differences in the relative change of standardized Δ^2^H, water potential and stomatal conductance at different treatment duration with Fisher’s LSD (*P* < 0.05). *P*-value represents the significance in average of root water uptake between conifers and broadleaved species at 20 days acclimation tested with TukeyHSD. *P*-values were neglected if no significance (*P* > 0.05) between conifers and broadleaved species for average water uptake, leaf water potential. Data points represent the mean values across species within each functional type ± s.e. (values for the individual species are presented in Figure S3 available as Supplementary data at *Tree Physiology* Online).

Water uptake and transport as indicated by changes of ^2^H-labels in leaf water declined over the 20 days exposure to 2 °C and 7 °C relative to 15 °C root temperature, with immediately stronger restriction for herbs than tree species (Figure 1A and B). At 7 °C root temperature, the water transport relative to 15 °C was only moderately reduced by ⁓10% in broadleaved trees and conifers immediately after transfer to the water bath (day 0) and showed a decline by another ca 15% after 10 days in both functional tree types (significant only for conifers, Figure 1A). After 20 days, the water uptake further decreased significantly in broadleaved trees and conifers, with a much stronger decline in broadleaved trees than in conifers (Figure 1A).

At 2 °C root temperatures, both conifers and broadleaved trees showed significant declines of water uptake relative to 15 °C over time form a ca 20% reduction at day 0 to ca 70% reduction at day 10 (Figure 1B).The further decline between day 10 and day 20 was not significant in both tree functional types (Figure 1B). By comparison, the average rate of water uptake in the two herbs rapidly declined after root exposure to cold temperatures (day 0), with ca 70% reduction at 7 °C and ca 85% reduction at 2 °C, and remained at this low level throughout the 20 days of the experiment (Figure 1A and B). The absolute ^2^H label changes in leaf water for the individual species for all root temperatures and days underlying the above calculations are shown in Figure S1 and S2 available as Supplementary data at *Tree Physiology* Online. The species specific changes of water uptake relative to 15 °C are presented in Figure S5A and B available as Supplementary data at *Tree Physiology* Online.

To further quantify the negative effects of low root temperatures of 2 °C and 7 °C on other plant physiological parameters, we also calculated the relative change of mid-day leaf water potentials for three functional plant groups (Figure 1C and D) and leaf stomatal conductance for broadleaved trees and herbs (Figure 1E and F, see Figure S4–S6 available as Supplementary data at *Tree Physiology* Online for the species-specific underlying values). Averaged across functional plant types, broadleaved and conifer trees exposed to 7  °C root temperature both revealed initially only moderate leaf water potential reduction, but broadleaved trees showed a stronger decline over the 20 days period than conifers (Figure 1C). At 2 °C root temperature, the initial negative effect on leaf water potentials was stronger than at 7 °C for broadleaved and conifer trees, and the water potentials decreased continuously and significantly over time in both functional tree types (Figure 1D). Unlike for the water uptake rates, the two herbs revealed an initial drop of their plant water potentials similar to trees (day 0) at 7 °C and 2 °C root temperature, but at both temperatures they then showed slightly stronger declines after 10 and 20 days than the two functional tree types (Figure 1C and D). Stomatal conductance was decreased at low root temperatures, with a stronger effect at 2 °C compared with 7 °C (Figure 1E and F) and decreased significantly at 7 °C and 2 °C over the 20 days exposure period in broadleaved trees, while on average the herbs had initially stronger reduced stomatal conductance with no significant changes over time. A full factorial ANOVA for species, root temperature and duration effects on water uptake, water potential and stomatal conductance revealed significant effects for most single factors, but no significant interactions except a marginally significant species x duration effect on water potentials (Table 2).

**Table 2:** Full-factorial ANOVA for the effects of tree species, treatment duration (Day) and root Temperature (temperature) on the relative change in standardized Δ^2^H (after 6 h of ^2^H-H_2_O labeling of per acclimatization day), stem water potential, stomatal conductance(only for broadleaved species).

	Relative change∆ ^2^H	Relative change in stem water potential		Relative change in stomatal conductance		
	F	P	F	P	F	P
Species	1.08	0.43	3.972	**0.01**	3.785	0.0434
Temperature	30.08	<**0.001**	50.39	<**0.001**	35.486	**<0.001**
Day	97.51	<**0.001**	44.36	<**0.001**	28.476	**<0.001**
Species × Day	1.589	0.155	2.750	**0.043**	1.351	0.3375
Species × Temperature	0.418	0.904	0.759	0.654161	2.182	0.1517
Day × Temperature	0.286	0.601	2.364	0.146441	1.968	0.198221

Bold values indicate statistical significance at the P < 0.05 level.

### Temporal changes of water uptake and hydraulics at constantly low root temperatures in dependency of the natural upper distribution limit of tree species

**Figure 2:**
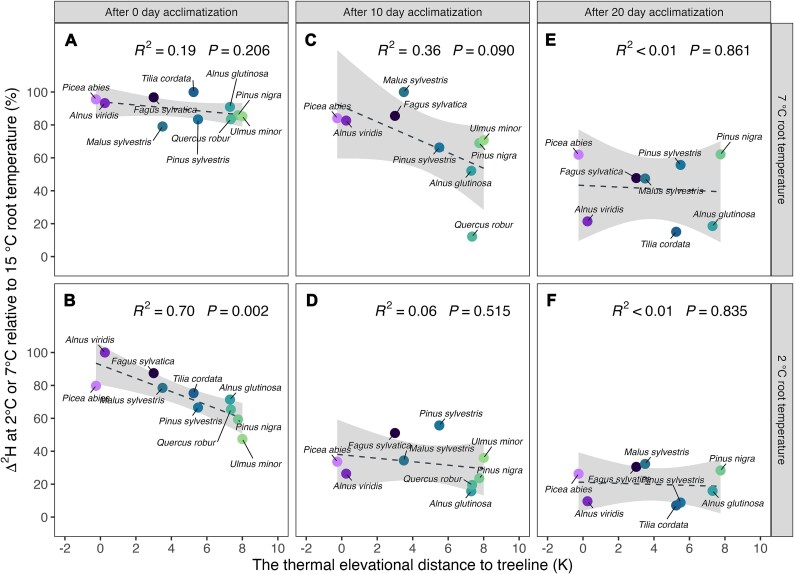
Relationships between the relative change of water ^2^H labels uptake in leaves (standardized Δ^2^H) and the species’ thermal distance to tree line at 7 °C (A, C and E) and 2 °C (B, D and F) root temperature at 6 h of source water ^2^H-pulse labeling at 0, 10 and 20 acclimatized days, respectively. The dotted fit lines are linear correlations, the gray area represents the 95% confidence interval.

Our tree species selection for this study aimed to cover a wider range of natural elevational distribution limits among species, including species that reach the alpine treeline as well as species that have their elevational limit over 1000 m below the treeline (Table 1). It allowed us to test if the change of water uptake speed with increasing duration of the low temperature stress is dependent on the species’ natural cold temperature distribution limits. Therefore, we plotted the change of water uptake at 7 °C or 2  °C relative to 15 °C root temperature at 0, 10 and 20 days exposure time for each tree species against its high elevation distribution limit, given as the thermal distance to treeline (Figure 2). The rate of water uptake in all tree species treated with 7 °C root temperature was only moderately reduced at day 0, independent of their thermal elevational range limits (Figure 2A). After 10 days exposure to 7 °C, however, there was a moderate correlation with the species-specific thermal elevational limits (R^2^ = 0.36, *P* = 0.090), with smaller reductions in upper montane species and larger reductions in lower montane species (Figure 2C). After 20 days, all species had strongly reduced their rate of water uptake irrespective of their thermal elevational range (Figure 2E). In contrast, at 2 °C root temperatures, there was a significant correlation (R^2^ = 0.70, *P* = 0.002) with the species’ upper elevational range limits immediately after transfer to the water bath (day 0, Figure 2B), were the rate of water uptake relative to the 15 °C treatment decreased stronger in species with lower elevational range limits. After 10 and 20 days exposure to 2 °C root temperature, water uptake rates decreased markedly in all species with no correlation to the species’ upper range limits (Figure 2D and F).

**Figure 3:**
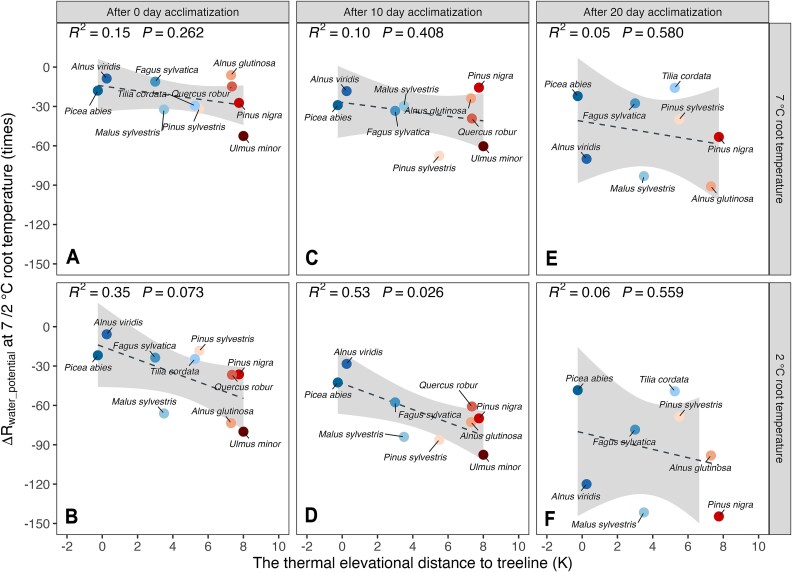
Relationships between the relative change of leaf water potential and the species’ thermal distance to tree line at 7 °C (A, C and E) and 2 °C (B, D and F) root temperature at 0, 10 and 20 acclimatized days, respectively. The dotted fit lines are linear correlations, the gray area represents the 95% confidence interval.

At 7 °C root temperature, no significant correlations were found between the tree species’ elevational range limits and their change of midday leaf water potential relative to 15 °C for any timepoint across the 20 days treatment (Figure 3). In seedlings exposed to 2 °C root temperature there were moderate correlations with the species upper elevation rang limits at day 0 (*P* = 0.073) and day 10 (*P* = 0.026) (Figure 3). After 20 days, most species had strongly reduced water potentials with no correlations with the species’ upper distribution limits.

### Effects of low root temperatures on the growth of tree seedlings

**Figure 4:**
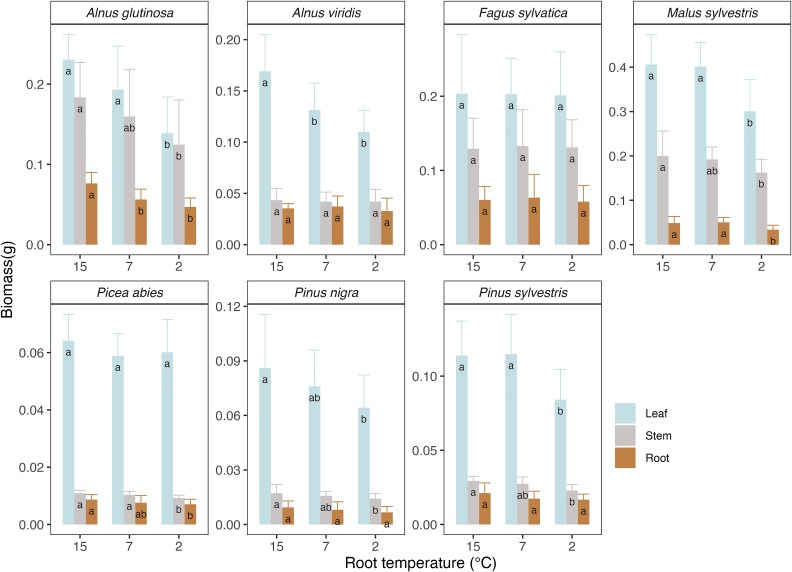
Biomass in different organs (root, stem, leaf) of individual investigated tree species after 20 days acclimatization. Lowercases represent the significant difference in biomass of same organs of individual species at three different root temperature treatments (15 °C, 7 °C and 2 °C) with Fisher’s LSD (*P* < 0.05). The mean values were averaged by 10 seedlings of each investigated species at least (*n* = 10 ± s.e.).

**Figure 5:**
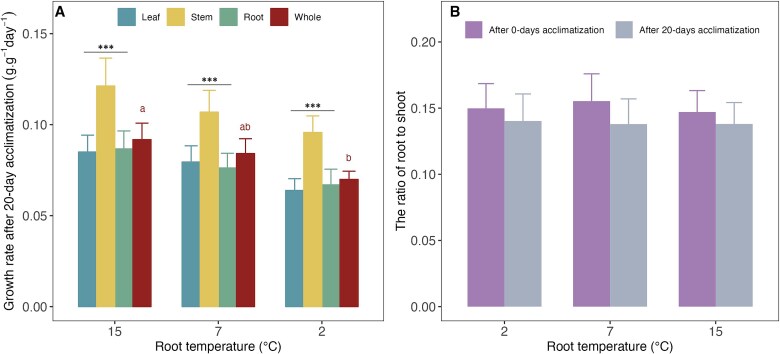
Relative growth rate of tree seedlings exposed to three different root temperatures (15 °C, 7 °C and 2 °C) at 20 days acclimatization compared with the acclimatization of 0-day (as showed the initial biomass at 0 days in Figure S7 available as Supplementary data at *Tree Physiology* Online) (A). The ratio of root to shoot at 0 and 20 days in seedlings treated with three different root temperatures (B). Lower cases letters represent the significant difference in average of growth rate of the whole plants across seven investigated tree species at three different root temperature treatments (15 °C, 7 °C and 2 °C) with Fisher’s LSD (*P* < 0.05). No letter represents no significant effects (*P* > 0.05) of different root temperatures on the growth rate of different organs (A) and of root to shoot ratios. Asterisks represent the significant difference in average of growth rate of stem and other organs (root and leaf) across seven investigated tree species tested with TukeyHSD (^***^P < 0.001). The values are means averaged across seven species with at least 10 seedlings per species ± s.e.

Among the seven tree species investigated for growth, the 20 days exposure to low root temperatures significant reduced root growth in three species (*Alnus glutinosa*, *Malus sylvestris* and *Pinus sylvestris*), stem growth in five species (all species except for *Alnus viridis* and *Fagus sylvatica*) and leaf growth in five species (all species except for *Picea abies* and *Fagus sylvatica*) (Figure 4). When calculating the relative growth rates (RGR, biomass increase after 20 days relative to the initial biomass at day 0 shown in Figure S7 available as Supplementary data at *Tree Physiology* Online) for different tree organs across all species, we found a significant decrease of the RGR across all organs, and similar but non-significant trends for leaves, stems and roots with decreasing root temperature (Figure 5A). As a consequence, the ratio of root to shoot were not significantly changed by the root temperature, implying that the 20 days exposure to low root temperatures led to a similar growth reduction of below and aboveground plant tissues (Figure 5B).

## Discussion

Our results confirmed previous experimental evidence that low root temperatures cause significant restrictions of water uptake and transport from roots to leaves and provided new evidence that there is no acclimation to low root temperatures toward improved water uptake over the treatment time. Low root temperatures also resulted in similar reductions of below- and aboveground plant growth. At the beginning of the experiment, tree species showed less reduction in root water uptake compared with the two more warm adapted herbaceous species, but the water uptake of all tree species steadily declined toward the same low level of the herbs after 20 days. Most interestingly, the temperature sensitivity of root water uptake for trees tended to correlate with the natural cold limits of the species only in the initial phase of the experiment at 2 °C, but not at 7 °C root temperature. Against our initial hypothesis, there were no adjustments to improve water uptake after longer cold root exposure, but a uniform decline to similarly low rates across all species and functional plant types.

Low root-zone temperature is a well-known abiotic stress that inhibits plant water transport from roots to leaves more than can be expected from the purely physical effect of increasing water viscosity alone (Kaufmann 1975, Cochard et al. 2000, Wan et al. 2001). The main reason is related to an exponentially increasing resistance against the radial symplastic water movement through bio-membranes of the root cortex and endodermis, especially at temperatures below 15 °C (Javot and Maurel 2002, Ehlert et al. 2009). Water can be transported through lipid bilayers either via direct diffusion, or (more efficiently) via osmotic transport through aquaporins (McElrone et al. 2007, Maurel et al. 2015, Maurel and Nacry 2020). Both pathways have been shown to be restricted by low temperatures in different plant groups, including temperate trees (Wan et al. 1999, Maurel 2007, Barrero-Sicilia et al. 2017, Kapilan et al. 2018). Species-specific differences in the cold-sensitivity of root water uptake and transport are likely caused by differences in the properties of the bio-membranes as well as in the abundance and isoform compositions of aquaporins (Lee and Chung 2005, Ranganathan et al. 2016, Kapilan et al. 2018). Consequently, it can also be expected that plants might acclimatize to low root temperatures by structural adjustments of the lipid membranes and changes in the abundance and composition of aquaporins Aroca et al. (2012). However, our knowledge on root conductance acclimation to low temperatures is very limited, and only a few studies have so far experimentally investigated changes of plant hydraulics at continuous low root temperatures over longer time periods.

In our current study, we did not find indications for physiological adjustments to improve water uptake induced by cold root temperatures over time in any of the investigated species. Rather, the capacity to take up water declined over the 20 days continuous exposure to low root temperatures, intrinsically reflecting the limited potential acclimation of species cold sensitivity controlled by aquaporins in root cell membranes. Wan et al. (1999) investigated the effect of cold root temperatures on root hydraulic conductance of *Populus tremuloides* seedlings along 28 days of continuous treatment. In their study, they found an immediately very strong restriction of root water flow at 5 °C root temperature that remained unchanged along the 28-day period, while they found indications that at 10 °C the initial stark restriction of root water transport might improve toward the end of the experiment. While there were no improvements for water uptake over time in any of the investigated species in the current study, we found differences with respect to the dynamic of root water uptake and transport changes among the investigated functional groups. Both warm-temperate, annual herbs showed immediate and very strong declines of water uptake that largely remained unchanged throughout the experiment already at 7 °C root temperature. In contrast, the rate of root water uptake remained initially high in all tree species but gradually declined over the 20 days treatments at the two low temperatures, with a significantly faster decline of broadleaved trees compared with conifers at 7 °C, but not at 2 °C. A persistant cold-limitation of root respiration might be a plausible explanation for the observed decreasing water uptake over time. On one hand, it can limit the synthesis of plasma membrane aquaporins (Martre et al. 2002, Hachez et al. 2006, Lee et al. 2012). On the other hand, low temperatures might directly cause the inactivation of already existing aquaporins (Lee et al. 2005) accompanied by an additionally restriction of osmotic regulation over mebranes (Ye et al. 2004). Besides, cold root temperatures can also lead to a substantial accumulation of hydrogen peroxide (H_2_O_2_) which damages the structure of cell membranes and impedes plant metabolic processes (Aroca et al. 2005). Cold-tolerant plants can react to cold-induced accumulation of H_2_O_2_ with an increased production of peroxidases (PODs) to sustain cellular structure and function (He et al. 2018). The annual herbs (maize and sunflower) investigated in this study might lack such cold acclimation mechanisms in contrast to the investigated temperate tree species that initially could keep up considerable water uptake capacities at cold root temperatures.

The continuous reduction of root water uptake and transport in the tree species over time was also reflected in declining leaf water potentials in our study, which is consistent with previous finding among different tree species (Wang and Hoch 2022, Li and Hoch 2024). Interestingly, for the two observed herbs of this study, the faster and stronger drop of water uptake was initially not accompanied by the larger reductions of leaf water potentials. The initially stronger stomatal regulation in herbs compared with broad-leaved trees might partially explain this seemingly contradicting result. The other possible reason can be attributed to foliar water storage that might have compensated the immediate lack of water supply at the beginning of the low root temperature stress with additional strong stomatal regulations (Ishii et al. 2014, Chin et al. 2022). This drought-like water deficit caused by low root-zone temperature is likely a contributing factor for the limitation of plant growth by restricted cell enlargement and differentiation at reduced water turgor pressure of growing plant cells (Woodruff and Meinzer 2011, Peters et al. 2021). In addition, reduced leaf gas exchange and carbon assimilation due to decreased water supply at cold root temperatures might further amplify the direct hydraulic restrictions of plant growth (Delucia 1986, Lintunen et al. 2020, Wang and Hoch 2022).

Previous experiments with cooled roots showed fast and severe restrictions of root growth when temperatures decreased below 10 °C and extremely slow growth at temperatures below 5 °C (Alvarez-Uria and Körner 2007, Schenker et al. 2014). Within the current experiment as well as a previous study (Wang and Hoch 2022), it could be shown that, at least in the short-term, the growth restrictions are indeed proportional for below and aboveground tree organs, with no changes in the root:shoot ratio, even if only roots are cooled. This implies that the growth limitations might occur primarily due to cold-induced hydraulic constraints, limiting the turgor-driven cell expansion in all plant parts, rather than direct thermal limitations of cell growth processes, which would be largely restricted to the cooled roots. Cold growth limitations of below and aboveground tree tissue has been also found under natural conditions on a montane permafrost site in the Swiss Jura mountains, where over 100-year-old Norway spruce trees showed extremely reduced aboveground growth of shoots and needles at a seasonal mean soil temperature ⁓6 °C despite much warmer shoot conditions during the growing season (Körner and Hoch 2006, Hoch 2008). In a reciprocal root:shoot cooling experiment with potted conifer seedlings, Hoch (2013) also documented significant declines of growth after a whole growing season in all tree organs if only roots or shoots were cooled, although root growth did seem to be slightly more sensitive to low temperatures than aboveground parts in this longer-term study.

Interestingly, our study revealed a large variability of cold sensitivity of root water uptake and transport among tree species immediately after exposure to 2 °C root temperature, with lower montane species showing larger restrictions in root water uptake than species occurring naturally at higher elevations. As has been shown previously also by Li and Hoch (2024), at 7 °C root-zone temperature, such a dependency of a species’ cold sensitivity of root water uptake with its thermal elevational distribution limit was only established after 10 days at 7 °C root-zone temperature. The higher the upper elevational distribution limit of a tree species, the greater the species-specific tolerance to low temperature stress. However, irrespective of the underlying molecular mechanisms, our study revealed a striking difference between temperate trees and annual herbs with respect to their plant hydraulic constraints to cold stress. The perennial trees, with their higher cold tolerance, gradually reduced the capacity of root water uptake over time, while the annual, more cold-sensitive herbs showed immediate severe reductions of water uptake at cold root temperatures. As discussed above, the steady decline of water uptake capacity of temperate trees over the 20 days duration of the experiment might indicate accumulative low temperature stress. Alternatively, however, it might resemble a controlled process in temperate trees, where the water uptake capacity and hydraulic conductance is gradually reduced under persistently low root temperatures in preparation for the dormant winter seasons, when soil water availability can be significantly restricted or completely disabled in frozen soils (Mayr et al. 2006). Although physiological preparations of trees for winter dormancy, like cessation of cambial or meristematic activity and bud formation, are generally controlled by photoperiodism (Fracheboud et al. 2009), continuously low temperatures might also trigger these processes independent of day length (Gill et al. 2015, Brelsford et al. 2019). However, since our experimental setup did explicitly focus on low root temperatures while keeping aboveground conditions and photoperiod constant, it is likely that root hydraulic adjustments are co-controlled by decreasing temperatures and photoperiods and differ from the temporal dynamics found in this study. Nevertheless, the higher sensitivity against low temperatures in the lower elevation species, found in this study, could be explained by the fact that in their natural environment, these species are not experiencing longer periods with such cool soil temperatures during their native growing season. The 10 days of exposure to continuous 7 °C therefore might have initiated hydraulic adjustments for the dormant season in low elevation species, while for species that naturally can occur at or close to the alpine treeline, it took between 10 and 20 days to trigger this process. It is further interesting, that the investigated evergreen conifers reduced the water uptake capacity at cold root temperatures slower than the deciduous species, which might relate to their principal capability for photosynthesis also over winter.

In conclusion, our study revealed interesting species-specific differences among temperate trees and herbs with respect to longer-term effects of cold root temperatures on plant water uptake and growth. Against our initial hypothesis, none of the investigated species could improve its water uptake capacity at cold root temperatures over the 20 days of the experiment. However, the gradual decline of water uptake in temperate tree species over time, that was dependent of the species’ natural upper distribution limits, and was less severe for evergreen conifers, demonstrate that temperate trees can maintain high water conductivity over shorter cold spells during the growing season. One possible short-coming of our study relates to the fact that the seeds used for all tree species derived from populations situated at significantly lower elevations than the highest occurring populations of the respective species. Thus, we cannot exclude, that high elevation ecotypes might have improved root water uptake at low temperatures compared with the seedlings investigated here. Nevertheless, the question, if the decreasing water uptake capacity of trees over longer cold root exposure is related to accumulative stress, or if it is indicative of a controlled physiological downregulation of the trees’ hydraulic system in preparation for the winter season remains open and should be scrutinized in future investigations.

## Supplementary Material

Supplementary_final_tpaf018

## Data Availability

Data will be made available on request.

## References

[ref1] Alvarez-Uria P, Körner C (2007) Low temperature limits of root growth in deciduous and evergreen temperate tree species. Funct Ecol 21:211–218. 10.1111/j.1365-2435.2007.01231.x.

[ref2] Ameglio T, Morizet J, Cruiziat P, Martignac M, Bodet C, Raynaud H (1990) The effects of root temperature on water flux, potential and root resistance in sunflower. Agronomy 10:331–340. 10.1051/agro:19900407.

[ref3] Arndt CH (1937) Water absorption in the cotton plant as affected by soil and water temperatures. Plant Physiol 12:703–720. https://www.jstor.org/stable/40008125. 10.1104/pp.12.3.703. 16653440 PMC439324

[ref4] Aroca R, Amodeo G, Fernández-Illescas S, Herman EM, Chaumont F, Chrispeels MJ (2005) The role of aquaporins and membrane damage in chilling and hydrogen peroxide induced changes in the hydraulic conductance of maize roots. Plant Physiol 137:341–353. 10.1104/pp.104.051045. 15591439 PMC548864

[ref5] Aroca R, Porcel R, Ruiz-Lozano JM (2012) Regulation of root water uptake under abiotic stress conditions. J Exp Bot 63:43–57. 10.1093/jxb/err266. 21914658

[ref7] Bagnall D, Wolfe JO, King RW (1983) Chill-induced wilting and hydraulic recovery in mung bean plants. Plant Cell Environ 6:457–464. 10.1111/j.1365-3040.1983.tb01549.x.

[ref8] Barrero-Sicilia C, Silvestre S, Haslam RP, Michaelson LV (2017) Lipid remodelling: unravelling the response to cold stress in *Arabidopsis* and its extremophile relative *Eutrema salsugineum*. Plant Sci 1:194–200. 10.1016/j.plantsci.2017.07.017. PMC5567406 28818375

[ref9] Beck EH, Fettig S, Knake C, Hartig K, Bhattarai T (2007) Specific and unspecific responses of plants to cold and drought stress. J Biosci 32:501–510. 10.1007/s12038-007-0049-5. 17536169

[ref10] Bloom AJ, Zwieniecki MA, Passioura JB, Randall LB, Holbrook NM, St Clair DA (2004) Water relations under root chilling in a sensitive and tolerant tomato species. Plant Cell Environ 27:971–979. 10.1111/j.1365-3040.2004.01200.x.

[ref11] Brelsford CC, Nybakken L, Kotilainen TK, Robson TM (2019) The influence of spectral composition on spring and autumn phenology in trees. Tree Physiol 39:925–950. 10.1093/treephys/tpz026. 30901060

[ref12] Chin AR, Guzmán-Delgado P, Sillett SC, Kerhoulas LP, Ambrose AR, McElrone AR, Zwieniecki MA (2022) Tracheid buckling buys time, foliar water uptake pays it back: coordination of leaf structure and function in tall redwood trees. Plant Cell Environ 45:2607–2616. 10.1111/pce.14381. 35736139

[ref1c] Clements FE, Martin EV (1934) Effect of soil temperature on transpiration in Helianthus annuus. Plant Physiol 9:619. 10.1104/pp.9.3.619. PMC439085 16652901

[ref13] Cochard H, Martin R, Gross P, Bogeat-Triboulot MB (2000) Temperature effects on hydraulic conductance and water relations of *Quercus robur* L. J Exp Bot 51:1255–1259. 10.1093/jxb/51.348.1255. 10937701

[ref14] Cosgrove DJ (1993) Water uptake by growing cells: an assessment of the controlling roles of wall relaxation, solute uptake, and hydraulic conductance. Int J Plant Sci 154:10–21. 10.1086/297087. 11537965

[ref15] Dang QL, Cheng S (2004) Effects of soil temperature on ecophysiological traits in seedlings of four boreal tree species. For Ecol Manage 194:379–387. 10.1016/j.foreco.2004.03.004.

[ref16] Day TA, Heckathorn SA, DeLucia EH (1991) Limitations of photosynthesis in *Pinus taeda* L. (loblolly pine) at low soil temperatures. Plant Physiol 96:1246–1254. 10.1104/pp.96.4.1246. 16668326 PMC1080922

[ref17] Delucia EH (1986) Effect of low root temperature on net photosynthesis, stomatal conductance and carbohydrate concentration in Engelmann spruce (*Picea Engelmannii* Parry ex Engelm.) seedlings. Tree Physiol 2:143–154. 10.1093/treephys/2.1-2-3.143. 14975849

[ref18] Ehlert C, Maurel C, Tardieu F, Simonneau T (2009) Aquaporin-mediated reduction in maize root hydraulic conductivity impacts cell turgor and leaf elongation even without changing transpiration. Plant Physiol 150:1093–1104. 10.1104/pp.108.131458. 19369594 PMC2689965

[ref19] Fennell A, Markhart AH (1998) Rapid acclimation of root hydraulic conductivity to low temperature. J Exp Bot 49:879–884. 10.1093/jxb/49.322.879.

[ref20] Fletcher LR, Scoffoni C, Farrell C, Buckley TN, Pellegrini M, Sack L (2022) Testing the association of relative growth rate and adaptation to climate across natural ecotypes of *Arabidopsis*. New Phytol 236:413–432. 10.1111/nph.18369. 35811421

[ref21] Fracheboud Y, Luquez V, Bjorken L, Sjodin A, Tuominen H, Jansson S (2009) The control of autumn senescence in European aspen. Plant Physiol 149:1982–1991. 10.1104/pp.108.133249. 19201914 PMC2663763

[ref22] Gill AL, Gallinat AS, Sanders-DeMott R, Rigden AJ, Short Gianotti DJ, Mantooth JA, Templer PH (2015) Changes in autumn senescence in northern hemisphere deciduous trees: a meta-analysis of autumn phenology studies. Ann Bot 116:875–888. 10.1093/aob/mcv055. 25968905 PMC4640124

[ref23] Grossnickle SC (1988) Planting stress in newly planted jack pine and white spruce. Factors influencing water uptake. Tree Physiol 4:85–97. 10.1093/treephys/4.1.85. 14972837

[ref24] Hachez C, Zelazny E, Chaumont F (2006) Modulating the expression of aquaporin genes in planta: a key to understand their physiological functions? BBA Biomembranes 1758:1142–1156. 10.1016/j.bbamem.2006.02.017. 16580626

[ref25] Hales S (1727) Vegetable staticks or, an account of some statical experiments on the sap in vegetables: Being an essay towards a natural history of vegetation. W. & J. Innys, T. Woodward., London, UK. 10.3931/e-rara-43498.

[ref26] He WD, Gao J, Dou TX et al. (2018) Early cold-induced peroxidases and aquaporins are associated with high cold tolerance in Dajiao (*Musa* spp.‘Dajiao’). Front Plant Sci 9:1–18. 10.3389/fpls.2018.00282. 29568304 PMC5852111

[ref27] Hoch G (2008) The carbon supply of *Picea abies* trees at a Swiss montane permafrost site. Plant Ecol Divers 1:13–20. 10.1080/17550870802230791.

[ref28] Hoch G (2013) Reciprocal root-shoot cooling and soil fertilization effects on the seasonal growth of two treeline conifer species. Plant Ecol Divers 6:21–30. 10.1080/17550874.2011.643324.

[ref30] Ishii HR, Azuma W, Kuroda K, Sillett SC (2014) Pushing the limits to tree height: could foliar water storage compensate for hydraulic constraints in *Sequoia sempervirens*? Funct Ecol 28:1087–1093. 10.1111/1365-2435.12284.

[ref31] Javot H, Maurel C (2002) The role of aquaporins in root water uptake. Ann Bot 90:301–313. 10.1093/aob/mcf199. 12234142 PMC4240399

[ref32] Kapilan R, Vaziri M, Zwiazek JJ (2018) Regulation of aquaporins in plants under stress. Biol Res 51:4–1. 10.1186/s40659-018-0152-0. 29338771 PMC5769316

[ref33] Kaufmann MR (1975) Leaf water stress in Engelmann spruce: influence of the root and shoot environments. Plant Physiol 56:841–844. 10.1104/pp.56.6.841. 16659406 PMC541936

[ref1k] Körner C, Hoch G (2006) A test of treeline theory on a montane permafrost island. Arct. Antarct. Alp Res 38:113–119. 10.1657/1523-0430(2006)038[0113:ATOTTO]2.0.CO;2.

[ref34] Körner C (2021) The cold range limit of trees. Trends Ecol Evol 36:979–989. 10.1016/j.tree.2021.06.011. 34272073

[ref35] Kramer PJ (1942) Species differences with respect to water absorption at low soil temperatures. Am J Bot 29:828. 10.2307/2437650.

[ref36] Kuiper PJ (1964) Water uptake of higher plants as affected by root temperature. Veenman. 1–11. 10.2307/2437650.

[ref39] Lee SH, Singh AP, Chung GC (2004) Rapid accumulation of hydrogen peroxide in cucumber roots due to exposure to low temperature appears to mediate decreases in water transport. J Exp Bot 55:1733–1741. 10.1093/jxb/erh189. 15208334

[ref38] Lee SH, Chung GC, Steudle E (2005) Gating of aquaporins by low temperature in roots of chilling-sensitive cucumber and chilling-tolerant figleaf gourd. J Exp Bot 56:985–995. 10.1093/jxb/eri092. 15734792

[ref37] Lee SH, Chung GC, Jang JY, Ahn SJ, Zwiazek JJ (2012) Overexpression of PIP2; 5 aquaporin alleviates effects of low root temperature on cell hydraulic conductivity and growth in *Arabidopsis*. Plant Physiol 159:479–488. 10.1104/pp.112.194506. 22434042 PMC3375980

[ref40] Li Y, Hoch G (2024) The sensitivity of root water uptake to cold root temperature follows species-specific upper elevational distribution limits of temperate tree species. Plant Cell Environ 47:2192–2205. 10.1111/pce.14874. 38481108

[ref42] Lintunen A, Paljakka T, Salmon Y, Hölttä T (2018) Belowground hydraulic conductance in a mature boreal scots pine tree. Acta Hortic 1222:103–108. 10.17660/ActaHortic.2018.1222.14.

[ref41] Lintunen A, Paljakka T, Salmon Y, Dewar R, Riikonen A, Hölttä T (2020) The influence of soil temperature and water content on belowground hydraulic conductance and leaf gas exchange in mature trees of three boreal species. Plant Cell Environ 43:532–547. 10.1111/pce.13709. 31873942

[ref43] Lippu J, Puttonen P (1991) Soil temperature limitations on gas exchange in 1-year-old *Pinus sylvestris* (L) seedlings. Scand J Forest Res 6:73–78. 10.1080/02827589109382650.

[ref44] Liu C, Peltoniemi M, Alekseychik P, Mäkelä A, Hölttä T (2024) A coupled model of hydraulic eco-physiology and cambial growth—accounting for biophysical limitations and phenology improves stem diameter prediction at high temporal resolution. Plant Cell Environ 48:1344–1365. 10.1111/pce.15239. 39449245 PMC11695789

[ref45] Martre P, Morillon R, Barrieu F, North GB, Nobel PS, Chrispeels MJ (2002) Plasma membrane aquaporins play a significant role during recovery from water deficit. Plant Physiol 130:2101–2110. 10.1104/pp.009019. 12481094 PMC166722

[ref46] Maurel C (2007) Plant aquaporins: novel functions and regulation properties. FEBS Lett 581:2227–2236. 10.1016/j.febslet.2007.03.021. 17382935

[ref47] Maurel C, Nacry P (2020) Root architecture and hydraulics converge for acclimation to changing water availability. Nat Plants 6:744–749. 10.1038/s41477-020-0684-5. 32601421

[ref48] Maurel C, Boursiac Y, Luu DT, Santoni V, Shahzad Z, Verdoucq L (2015) Aquaporins in plants. Physiol Rev 95:1321–1358. 10.1152/physrev.00008.2015. 26336033

[ref49] Mayr S, Hacke U, Schmid P, Schwienbacher F, Gruber A (2006) Frost drought in conifers at the alpine timberline: xylem dysfunction and adaptations. Ecology 87:3175–3185. 10.1890/0012-9658(2006)87[3175:FDICAT]2.0.CO;2. 17249241

[ref50] McElrone AJ, Bichler J, Pockman WT, Addington RN, Linder CR, Jackson RB (2007) Aquaporin-mediated changes in hydraulic conductivity of deep tree roots accessed via caves. Plant Cell Environ 30:1411–1421. 10.1111/j.1365-3040.2007.01714.x. 17897411

[ref51] Melkonian J, Yu LX, Setter TL (2004) Chilling responses of maize (*Zea mays* L.) seedlings: root hydraulic conductance, abscisic acid, and stomatal conductance. J Exp Bot 55:1751–1760. 10.1093/jxb/erh215. 15235000

[ref52] Mellander PE, Bishop K, Lundmark T (2004) The influence of soil temperature on transpiration: a plot scale manipulation in a young scots pine stand. For Ecol Manage 195:15–28. 10.1016/j.foreco.2004.02.051.

[ref53] Morin X, Augspurger C, Chuine I (2007) Process-based modeling of species distributions: what limits temperate tree species range boundaries? Ecology 88:2280–2291. 10.1890/06-1591.1. 17918406

[ref1m] Merrill RK (1975) Leaf water stress in Engelmann spruce: influence of the root and shoot environments. Plant Physiol 56:841–844. 10.1104/pp.56.6.841. PMC541936 16659406

[ref54] Murai-Hatano M, Kuwagata T, Sakurai J et al. (2008) Effect of low root temperature on hydraulic conductivity of rice plants and the possible role of aquaporins. Plant Cell Physiol 49:1294–1305. 10.1093/pcp/pcn104. 18676378

[ref55] Nagasuga K, Murai-Hatano M, Kuwagata T (2011) Effects of low root temperature on dry matter production and root water uptake in rice plants. Plant Prod Sci 14:22–29. 10.1626/pps.14.22.

[ref56] Nagelmüller S, Hiltbrunner E, Körner C (2017) Low temperature limits for root growth in alpine species are set by cell differentiation. AoB Plants 9:1–16. 10.1093/aobpla/plx054. PMC5710522 29218137

[ref57] Nievola CC, Carvalho CP, Carvalho V, Rodrigues E (2017) Rapid responses of plants to temperature changes. Temperature 4:371–405. 10.1080/23328940.2017.1377812. PMC5800372 29435478

[ref58] Pavel EW, Fereres E (1998) Low soil temperatures induced water deficits in olive (*Olea europaes*) trees. Physiol Plant 104:525–532. 10.1034/j.1399-3054.1998.1040402.x.

[ref59] Peters RL, Steppe K, Cuny HE, De Pauw DJ, Frank DC, Schaub M, Rathgeber CB, Cabon A, Fonti P (2021) Turgor–a limiting factor for radial growth in mature conifers along an elevational gradient. New Phytol 229:213–229. 10.1111/nph.16872. 32790914

[ref60] Pollock CJ, Tomos AD, Thomas A, Smith CJ, Lloyd EJ, Stoddart JL (1990) Extension growth in a barley mutant with reduced sensitivity to low temperature. New Phytol 115:617–623. 10.1111/j.1469-8137.1990.tb00493.x.

[ref61] R Core Team (2022) R: A language and environment for statistical computing. R Foundation for Statistical Computing, Vienna, Austria. https://www.R-project.org/.

[ref62] Randin CF, Paulsen J, Vitasse Y, Kollas C, Wohlgemuth T, Zimmermann NE, Körner C (2013) Do the elevational limits of deciduous tree species match their thermal latitudinal limits? Glob Ecol Biogeogr 22:913–923. 10.1111/geb.12040.

[ref63] Ranganathan K, El Kayal W, Cooke JE, Zwiazek JJ (2016) Responses of hybrid aspen over-expressing a PIP2; 5 aquaporin to low root temperature. J Plant Physiol 192:98–104. 10.1016/j.jplph.2016.02.001. 26895330

[ref64] Running SW, Reid CP (1980) Soil temperature influences on root resistance of *Pinus contorta* seedlings. Plant Physiol 65:635–640. 10.1104/pp.65.4.635. 16661254 PMC440398

[ref1s] Sachs J (1875) Textbook of Botany. English translation. Macmillan and Co, Oxford.

[ref65] Schenker G, Lenz A, Körner C, Hoch G (2014) Physiological minimum temperatures for root growth in seven common European broad-leaved tree species. Tree Physiol 34:302–313. 10.1093/treephys/tpu003. 24584221

[ref1v] Vesque J (1878) De l’influence de la température du sol sur l’absorption de l’eau par les racines. Ann Sci Nat Bot 4:169–201.

[ref66] Wan X, Landhäusser SM, Zwiazek JJ, Lieffers VJ (1999) Root water flow and growth of aspen (*Populus tremuloides*) at low root temperatures. Tree Physiol 19:879–884. 10.1093/treephys/19.13.879. 10562405

[ref67] Wan X, Zwiazek JJ, Lieffers VJ, Landhäusser SM (2001) Hydraulic conductance in aspen (*Populus tremuloides*) seedlings exposed to low root temperatures. Tree Physiol 21:691–696. 10.1093/treephys/21.10.691. 11446998

[ref68] Wang W, Hoch G (2022) Negative effects of low root temperatures on water and carbon relations in temperate tree seedlings assessed by dual isotopic labelling. Tree Physiol 42:1311–1324. 10.1093/treephys/tpac005. 35038338

[ref69] Wieser G, Grams TEE, Matyssek R, Oberhuber W, Gruber A (2015) Soil warming increased whole-tree water use of Pinus cembra at the treeline in the central Tyrolean alps. Tree Physiol 35:279–288. 10.1093/treephys/tpv009. 25737326 PMC4820648

[ref70] Woodruff DR, Meinzer FC (2011) Water stress, shoot growth and storage of non-structural carbohydrates along a tree height gradient in a tall conifer. Plant Cell Environ 34:1920–1930. 10.1111/j.1365-3040.2011.02388.x. 21722142

[ref71] Woodward FI (1990) The impact of low temperatures in controlling the geographical distribution of plants. Philos Trans R Soc B 326:585–593. 10.1098/rstb.1990.0033.

[ref72] Yan C, Takeuchi S, Qiu GY (2019) Soil warming affects sap flow responses to meteorological conditions for *Betula albosinensis* at a subalpine wetland in the edge of Northeast Qinghai–Tibet plateau. Ecohydrology 12:1–12. 10.1002/eco.2079.

[ref73] Ye Q, Wiera B, Steudle E (2004) A cohesion/tension mechanism explains the gating of water channels (aquaporins) in Chara internodes by high concentration. J Exp Bot 55:449–461. 10.1093/jxb/erh040. 14739267

[ref74] Zhu S, Chen H, Dai Y, Lu X, Shangguan W, Yuan H, Wei N (2021) Evaluation of the effect of low soil temperature stress on the land surface energy fluxes simulation in the site and global offline experiments. J Adv Model Earth Syst 13:1–17. 10.1029/2020MS002403.

