# Supplementary material for: Physiological adjustments of temperate tree species and herbs in response to low root temperatures
Source: Tree Physiol. 2025 Feb 4;45(3):tpaf018. doi: 10.1093/treephys/tpaf018 (PMC11922318; doi:10.1093/treephys/tpaf018)
Supplement: Supplementary_final_tpaf018 [file supplementary_final_tpaf018.docx]

**Supplementary**

Fig. S1


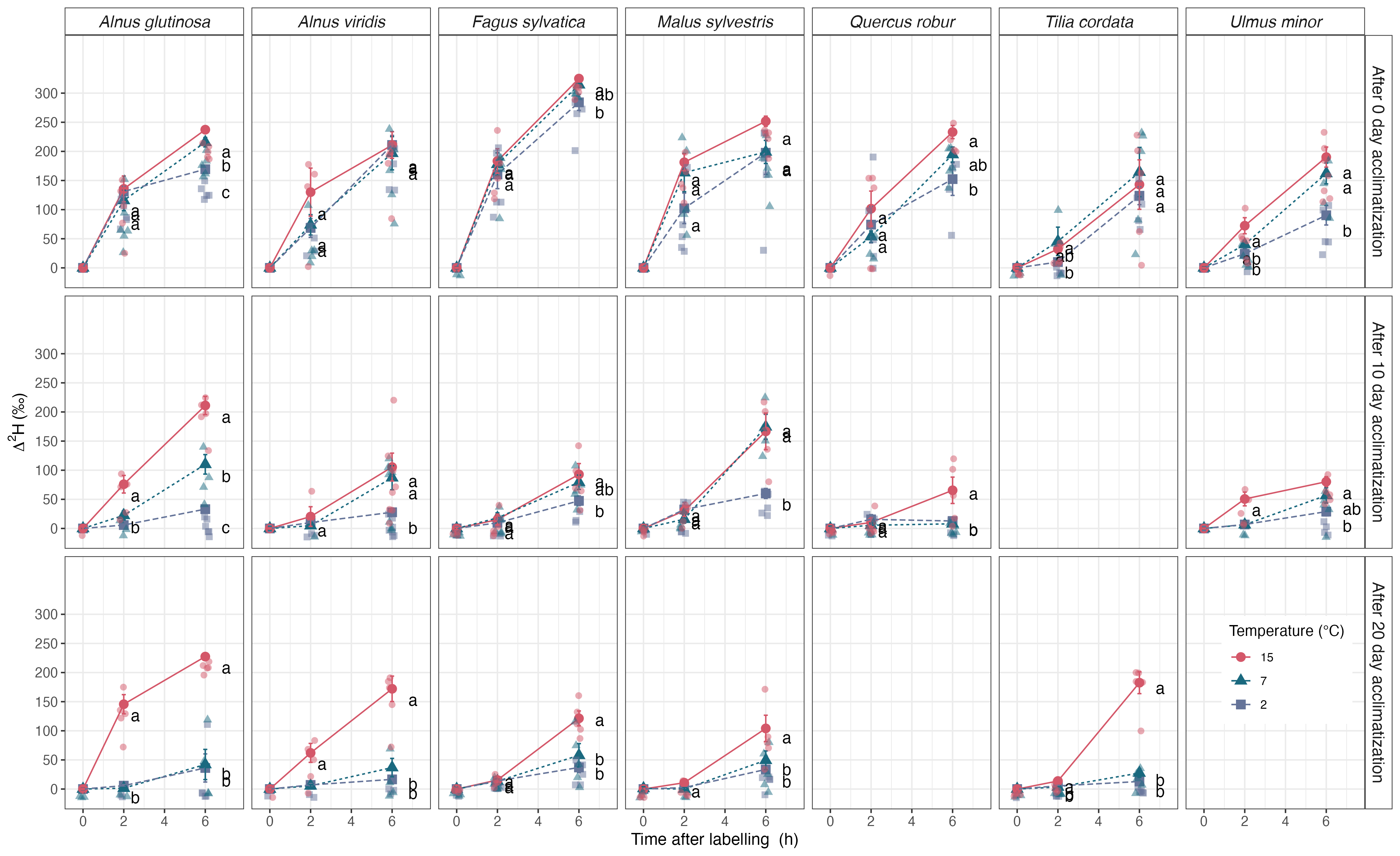


Fig. S1 Dynamic of leaf water Δ ^2^H uptake after 2 and 6h deuterium labelling in seven broadleaved tree seedlings exposed to different root temperature (15, 7, 2 °C) at 0 day, 10 days and 20 days acclimatization, respectively. ﻿The mean points of each species were averaged by 5 repetitions (light coloured points)﻿ at different root temperature after time point of pulse labelling (n=5±s.e.). Different lowercases indicate significant differences among the three temperature treatments for each species and time point tested with Fisher's LSD (*p* < 0.05).

Fig. S2


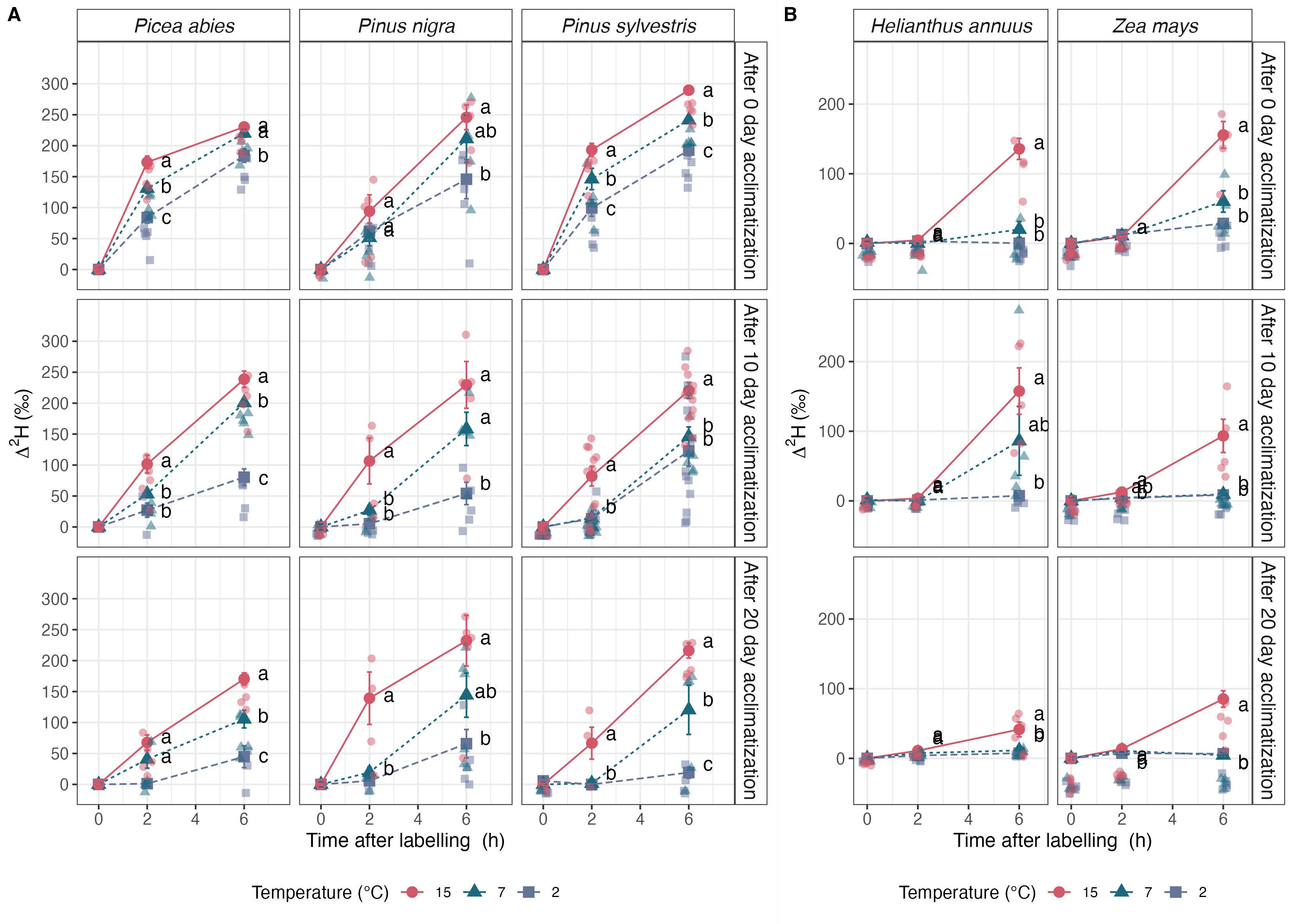


Fig. S2 Dynamic of leaf water Δ ^2^H uptake after 2 and 6h deuterium labelling in in three conifer (A) and two herbs (B) exposed to different root temperature (15, 7, 2 °C) at 0 day, 10 days and 20 days acclimatization, respectively. The mean points of each species were averaged by 5 repetitions (light coloured points)﻿ at different root temperature after time point of pulse labelling (n=5±s.e.). Different lowercases indicate significant differences among the three temperature treatments for each species and time point tested with Fisher's LSD (*p* < 0.05).

Fig. S3


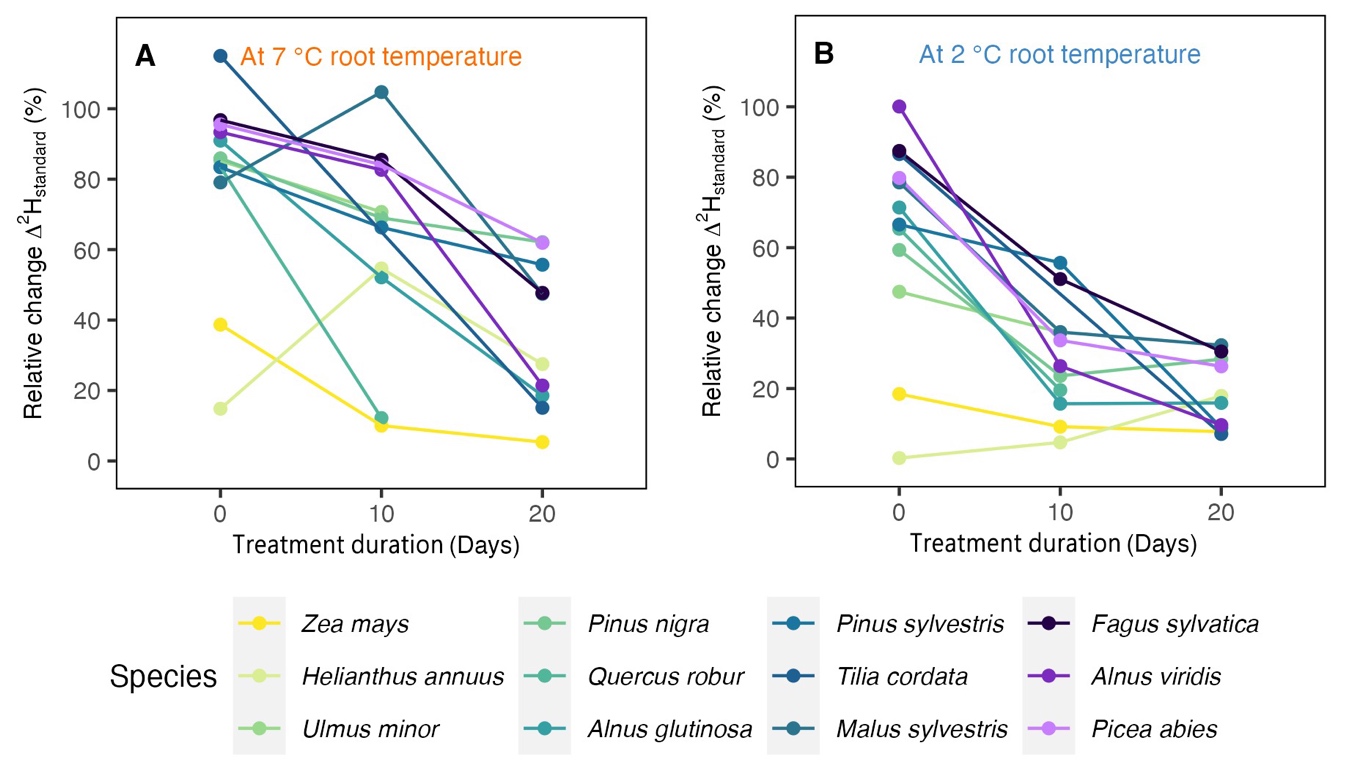


Fig. S3 Relative change of individually investigated species in Δ ^2^H in leaf water (at 6 h deuterium pulse labelling) in seedlings exposed to 7 °C (A) and 2 °C (B) relative to 15 °C root temperature along treatment duration (days).

Fig. S4


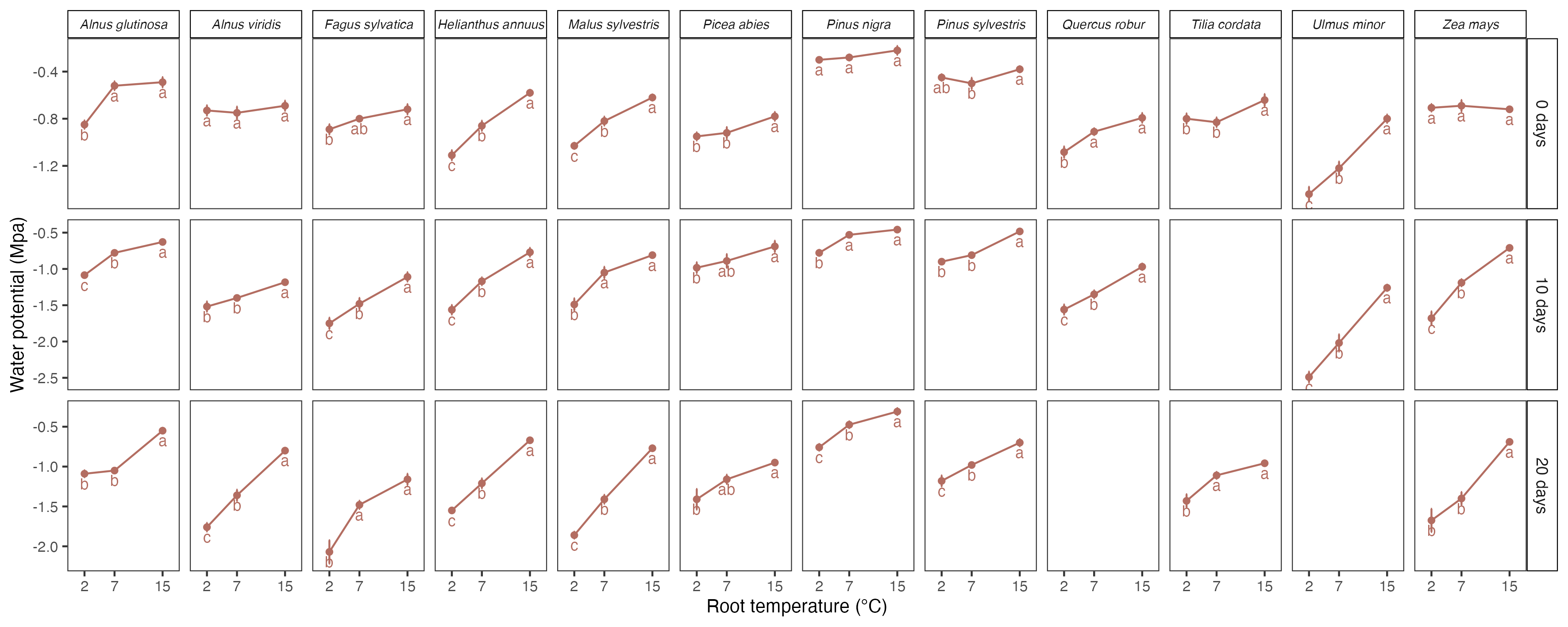


Fig. S4 Dynamic of leaf water potential in all investigated seedlings exposed to different root temperature (15, 7, 2 °C) at 0 day, 10 days and 20 days acclimatization, respectively. The mean points of each species were averaged by 5 repetitions at different root temperature after time point of pulse labelling (n=5±s.e.). Different lowercases indicate significant differences among the three temperature treatments for each species tested with Fisher's LSD (*p* < 0.05).

Fig. S5


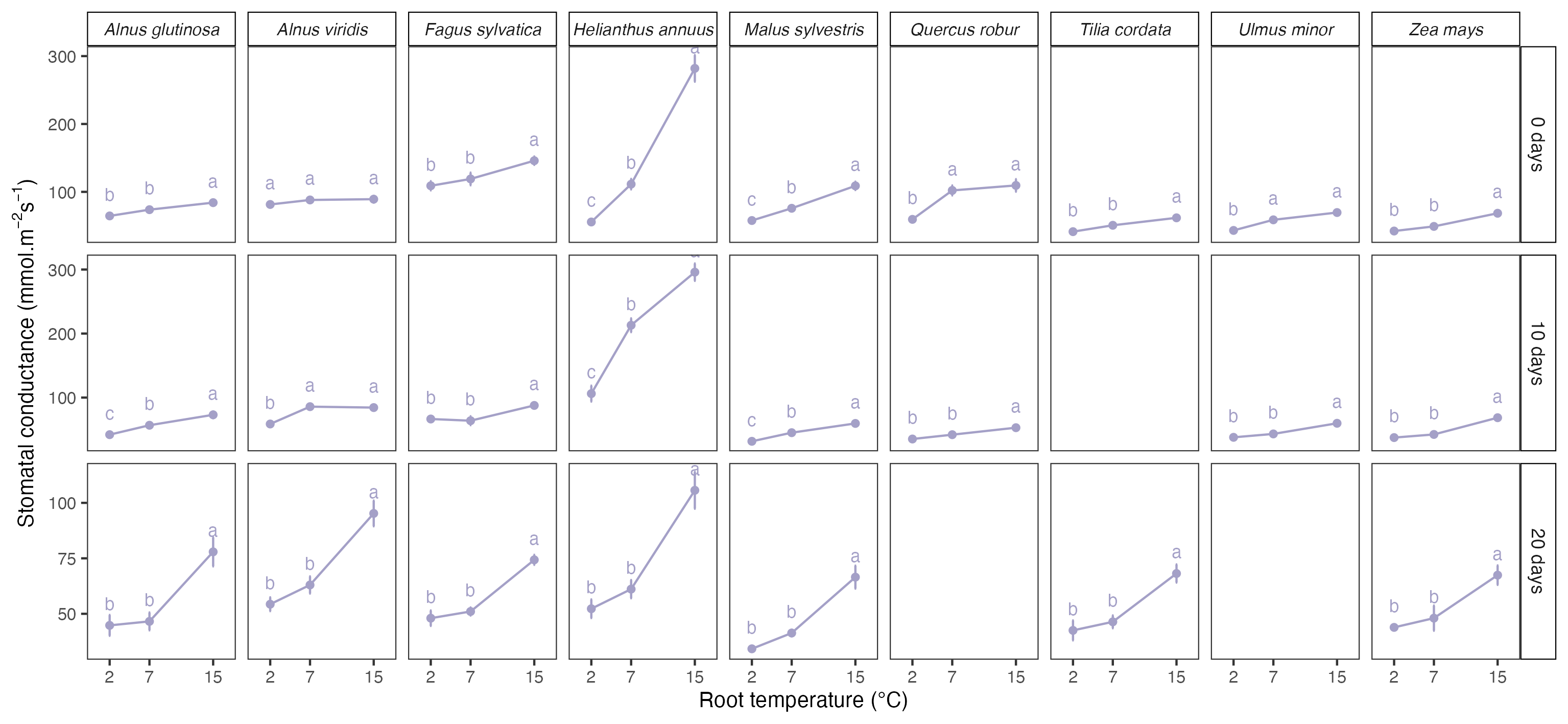


Fig. S5 Dynamic of stomatal conductance in broadleaved tree species and herbs seedlings exposed to different root temperature (15, 7, 2 °C) at 0 day, 10 days and 20 days acclimatization, respectively. The mean points of each species were averaged by 5 repetitions at different root temperature after time point of pulse labelling (n=5± s.e.). Different lowercases indicate significant differences among the three temperature treatments for each species tested with Fisher's LSD (*p* < 0.05).

Fig. S6


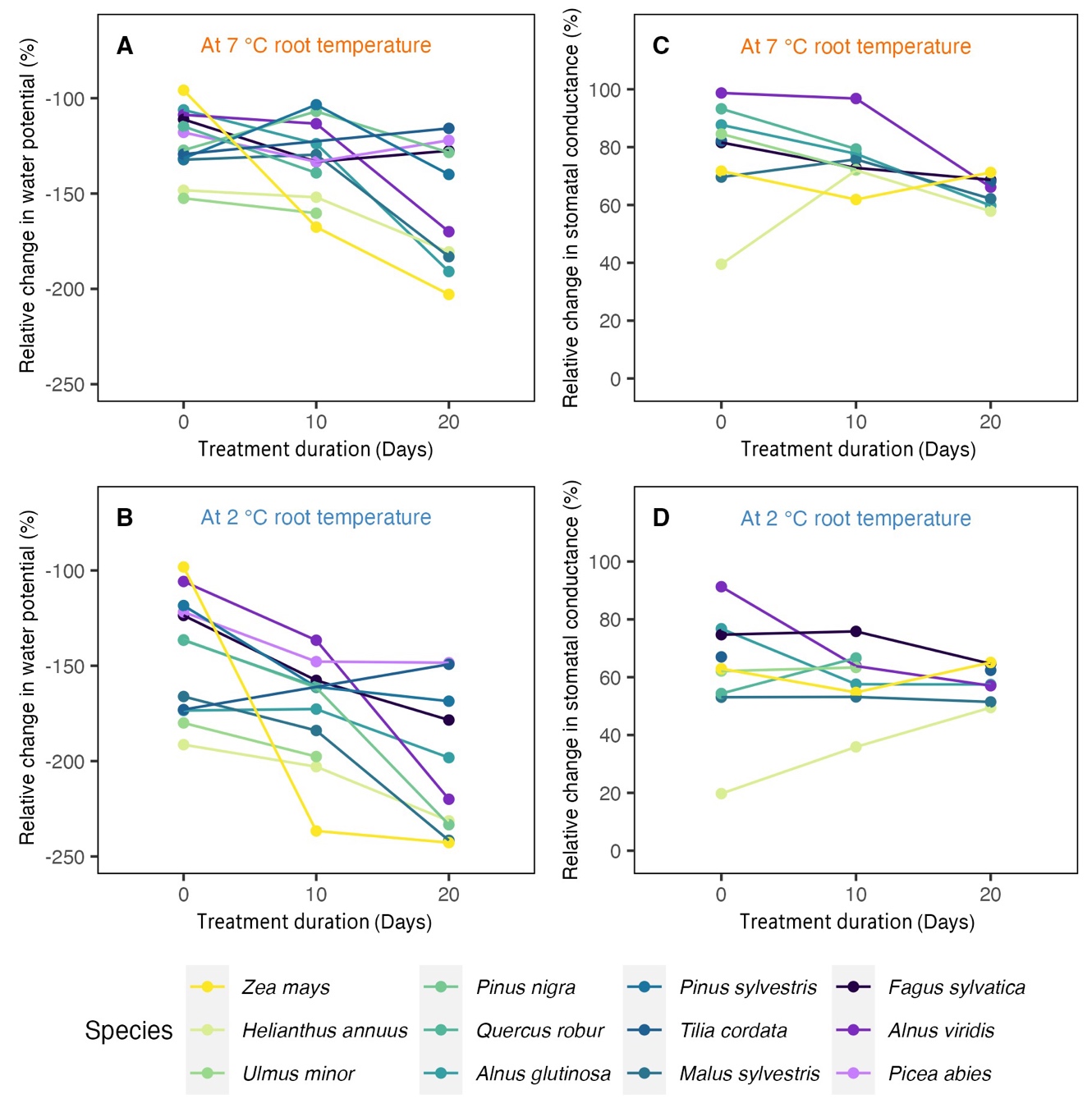


Fig. S6 Relative change of individually investigated species in stem water potential and stomatal conductance by exposure to 7 °C (A, C) and 2 °C (B, D) relative to 15 °C root temperature along treatment duration (days).

Fig. S7


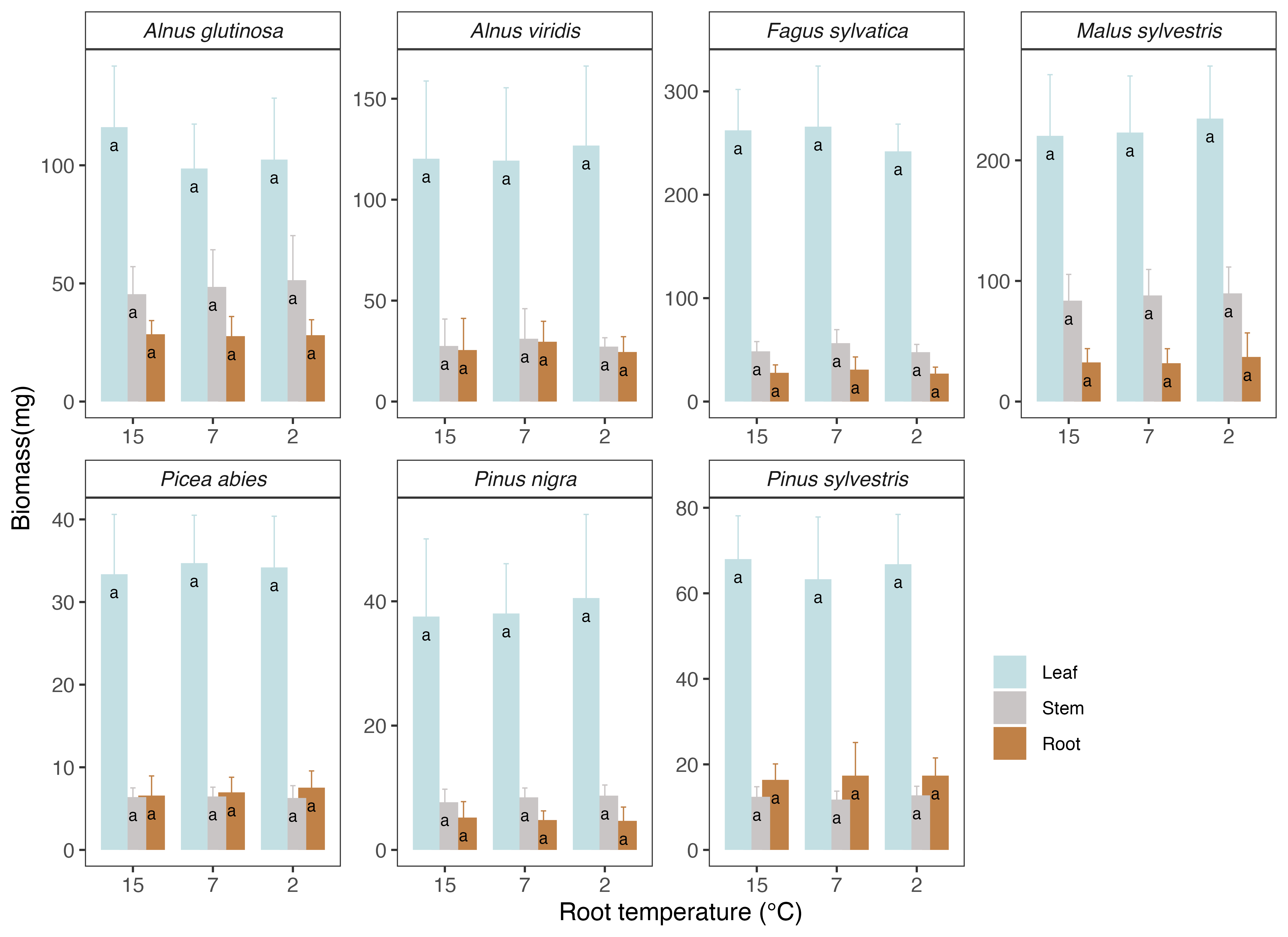


Fig. S7 Response of tree species root, stem and leaf biomass to different root temperatures (15, 7 and 2 °C) at 0 day acclimatization, respectively. The individual biomass of each species was averaged by 10 repetitions at least (n=10±s.e.). Different lowercases indicate significant differences among the three temperature treatments for the same tissues tested with Fisher's LSD (*p* < 0.05).
